# Global Trends in Phenylketonuria Treatment Research, 2000-2025: Bibliometric Analysis

**DOI:** 10.2196/90419

**Published:** 2026-09-03

**Authors:** Sitong Yang, Kaichao Song, Qingbo Chen, Jiandong Jiang, Lulu Wang

**Affiliations:** 1 State Key Laboratory of Bioactive Substance and Function of Natural Medicines, Institute of Medicinal Biotechnology, Chinese Academy of Medical Sciences & Peking Union Medical College, No. 1 Tiantan Xili, Dongcheng District, Beijing, Beijing, 100050, China, 86 13621284066; 2 Beijing Key Laboratory of Technology and Application for Anti-Infective New Drugs Research and Development, Institute of Medicinal Biotechnology, Chinese Academy of Medical Sciences & Peking Union Medical College, Beijing, Beijing, China

**Keywords:** phenylketonuria, PKU, bibliometric analysis, metabolic disorder, dietary management, precision medicine

## Abstract

**Background:**

Phenylketonuria (PKU) is the most common inborn error of amino acid metabolism, and if untreated, leads to severe neurocognitive impairment. Over the past 2 decades, treatment strategies have evolved from strict dietary phenylalanine restriction to include pharmacological therapies such as tetrahydrobiopterin and, more recently, enzyme substitution with pegvaliase. Despite these advances, significant heterogeneity exists in global research priorities, collaboration patterns, and the translation of emerging therapies into clinical practice. A systematic overview of the field’s development, thematic shifts, and remaining knowledge gaps is currently lacking.

**Objective:**

This study aimed to provide a systematic bibliometric analysis of global treatment research on PKU from 2000 to 2025. The aim was to quantify publication trends, collaboration patterns, thematic evolution, and research gaps, thereby informing future scientific and clinical directions.

**Methods:**

A search of the Web of Science Core Collection was performed on September 13, 2025. The search initially identified 1877 records. After screening, 1462 English-language articles and reviews were included. Publication trends were analyzed using Microsoft Excel (version 16.101), while VOSviewer 1.6.20 and CiteSpace 6.4R1 were used to visualize country- and institutional-level collaborations, journal networks, keyword co-occurrence, citation bursts, and thematic clusters. Statistical charts were generated with GraphPad Prism 10.2.1.

**Results:**

Annual publication output demonstrated an overall upward trend, peaking in 2022 with 111 publications. The United States led in both publication volume (375 studies) and total citations (11,142 citations), maintaining strong collaborative ties with several European countries, particularly the Netherlands and the United Kingdom. China ranked seventh globally in publication volume, although its citation impact remains comparatively limited. Key institutions, including the University of Groningen and Birmingham Children’s Hospital, as well as prominent scholars such as Francjan J van Spronsen and Anita MacDonald, have occupied central positions in the global PKU treatment research collaboration network over the study period. High-frequency and high-centrality keywords, such as “phenylalanine,” “dietary treatment,” and “tetrahydrobiopterin,” highlighted continued emphasis on metabolic control and targeted therapies. Keyword burst analysis revealed a gradual shift from conventional dietary management toward enzyme replacement therapies, investigations of neurocognitive outcomes, and precision medicine–oriented approaches.

**Conclusions:**

Over the past 25 years, PKU treatment research has progressed from foundational dietary interventions to molecular mechanistic studies and individualized therapeutic strategies. Future research should prioritize longitudinal multiomics investigations, targeted metabolic correction technologies, gene-based therapeutic approaches, and enhanced international collaboration, particularly to strengthen diagnosis and management capacities in low- and middle-income regions. Such efforts will be critical to advancing global standards of PKU care.

## Introduction

Inherited metabolic disorders comprise a group of rare conditions caused by pathogenic gene mutations that result in enzymatic defects and subsequent biochemical disturbances. These disorders can profoundly affect growth and overall quality of life in pediatric patients. Among them, amino acid metabolism defects hold particular importance, with phenylketonuria (PKU) being one of the most prevalent and representative conditions. Since its initial description, PKU has remained a focal topic in medical genetics, pediatrics, and nutritional science, with a global incidence estimated at approximately 1 in 10,000 live births [1].

PKU is an autosomal recessive disorder primarily caused by mutations in the phenylalanine hydroxylase (PAH) gene, which lead to markedly reduced or absent PAH enzyme activity in hepatocytes. This metabolic defect disrupts the normal conversion of phenylalanine to tyrosine, resulting in the pathological accumulation of phenylalanine in the blood, cerebrospinal fluid, and peripheral tissues. Alternative metabolites, such as phenylpyruvic acid, are excreted in urine, giving rise to the condition’s name. Elevated phenylalanine concentrations exhibit neurotoxic effects that severely impair brain development and function. Without timely treatment, affected individuals may develop progressive and irreversible intellectual disability, psychiatric and behavioral abnormalities, epilepsy, microcephaly, and other serious neurological impairments [2].

The global implementation of newborn screening programs and improved genetic disease management has substantially altered the natural history of PKU. Early diagnosis combined with lifelong dietary therapy can effectively prevent severe neurodevelopmental impairment [3]. However, as increasing numbers of patients reach adulthood and advanced age, new challenges have emerged, including long-term dietary adherence, psychosocial burdens, maternal PKU management, and adult-onset complications. These issues continue to expand and diversify the scope of PKU research.

Since the beginning of the 21st century, rapid advances in genomics, proteomics, and pharmaceutical development have transformed PKU research into a multidisciplinary knowledge system covering basic biomedical science, clinical pediatrics, nutrition, psychology, and health policy management. Given the dramatic growth of the literature, researchers, clinicians, and policymakers face increasing difficulty in understanding the field’s development trajectory, key knowledge structures, and evolving research priorities through traditional narrative reviews alone. Consequently, there is a pressing need for objective, quantitative approaches capable of providing macroscopic insights into the development of PKU research.

Bibliometrics, which applies quantitative statistical methods to academic publications, offers a powerful means of capturing research dynamics, identifying hotspots, mapping collaboration networks, and evaluating scientific influence within a given field [4,5]. Using tools such as co-occurrence analysis, cluster analysis, and collaboration network mapping, bibliometric methods transcend the limitations of individual studies and provide data-driven evidence to support scientific decision-making and strategic planning [6].

The 21st century represents a critical period for optimizing PKU management strategies and achieving breakthroughs in emerging therapies. However, to date, no comprehensive bibliometric analysis has systematically evaluated global PKU research over this period. To address this gap, this study conducts the first bibliometric assessment of PKU-related literature published between 2000 and 2025 in the Web of Science Core Collection (WoSCC). Through this analysis, the study aims to map the scientific knowledge landscape of the field and provide researchers with an objective, systematic reference framework.

Specifically, this study seeks to analyze annual publication trends, geographic distribution, and institutional contributions. Additionally, it aims to identify prolific authors, influential journals, and landmark publications; detect research hotspots and thematic evolution through keyword co-occurrence analysis; and demonstrate scientific collaboration networks to highlight global research linkages. By integrating these elements and discussing future research directions, this work aims to provide a comprehensive, data-driven overview of the development of PKU research. Therefore, this study aimed to systematically evaluate the global landscape of PKU treatment research from 2000 to 2025 using bibliometric methods.

## Methods

### Data Acquisition and Processing

A comprehensive literature search was performed on September 13, 2025, using the WoSCC, including both SCIE and SSCI editions. WoSCC was selected because it provides standardized bibliographic information and comprehensive citation data and is widely used in bibliometric studies. The search strategy used the following query in the “Topic” field:

(phenylketonuria OR “phenylalanine ketonuria” OR “Folling disease” OR (PKU NOT (“Peking University” OR “Beijing University”))) AND (therap* OR treat* OR manag* OR drug OR pharmacotherap* OR diet* OR sapropterin OR Pegvaliase OR Palynziq OR “case report” OR “clinical study” OR “clinical trial”) NOT (screening OR “newborn screen*” OR diagnos* OR detection OR assay OR biomarker).

The publication period was restricted from 2000 to 2025. Because the search was conducted on September 13, 2025, data for 2025 represent only a partial year. This strategy captured the major nomenclature and synonyms associated with PKU while excluding publications related to Peking University. The query focused on literature pertaining to disease management, dietary interventions, pharmacological treatments, and clinical studies, while excluding neonatal screening, diagnostic approaches, and biomarker research.

The initial search yielded 1877 records. Only English-language articles and reviews were retained for analysis. A total of 415 noneligible publications were excluded, including news items (n=4), data papers (n=1), retracted publications (n=3), corrections (n=12), book chapters (n=4), early access articles (n=4), letters (n=23), editorial materials (n=38), meeting abstracts (n=332), proceedings papers (n=48), book reviews (n=3), retractions (n=2), and reprints (n=1); the document-type categories listed are not mutually exclusive, because some records may be assigned to multiple document types. The final dataset consisted of 1462 publications, including 1272 articles and 190 reviews.

### Bibliometric Analysis and Visualization

A bibliometric analysis was conducted using Microsoft Excel (version 16.101), VOSviewer (version 1.6.20; Centre for Science and Technology Studies, Leiden University), and CiteSpace (version 6.4.R1; Drexel University) to generate a comprehensive overview of the research landscape. Microsoft Excel was used to compile annual publication counts from 2000 to 2025, thereby characterizing temporal trends in research productivity. VOSviewer was used to construct visual maps of international collaboration networks, co-authorship relationships, institutional partnerships, and journal co-citation patterns [7]. CiteSpace facilitated journal dual-map overlays, keyword co-occurrence analyses, reference co-citation networks, and burst-detection examinations, with outputs visualized as interpretable network diagrams [8]. The integrated application of these tools provided both quantitative and graphical insights into the intellectual structure, development tendency, and emerging research frontiers within the PKU field. Statistical charts were generated with GraphPad Prism 10.2.1 (GraphPad Software, Inc).

## Results

**Figure 1.**
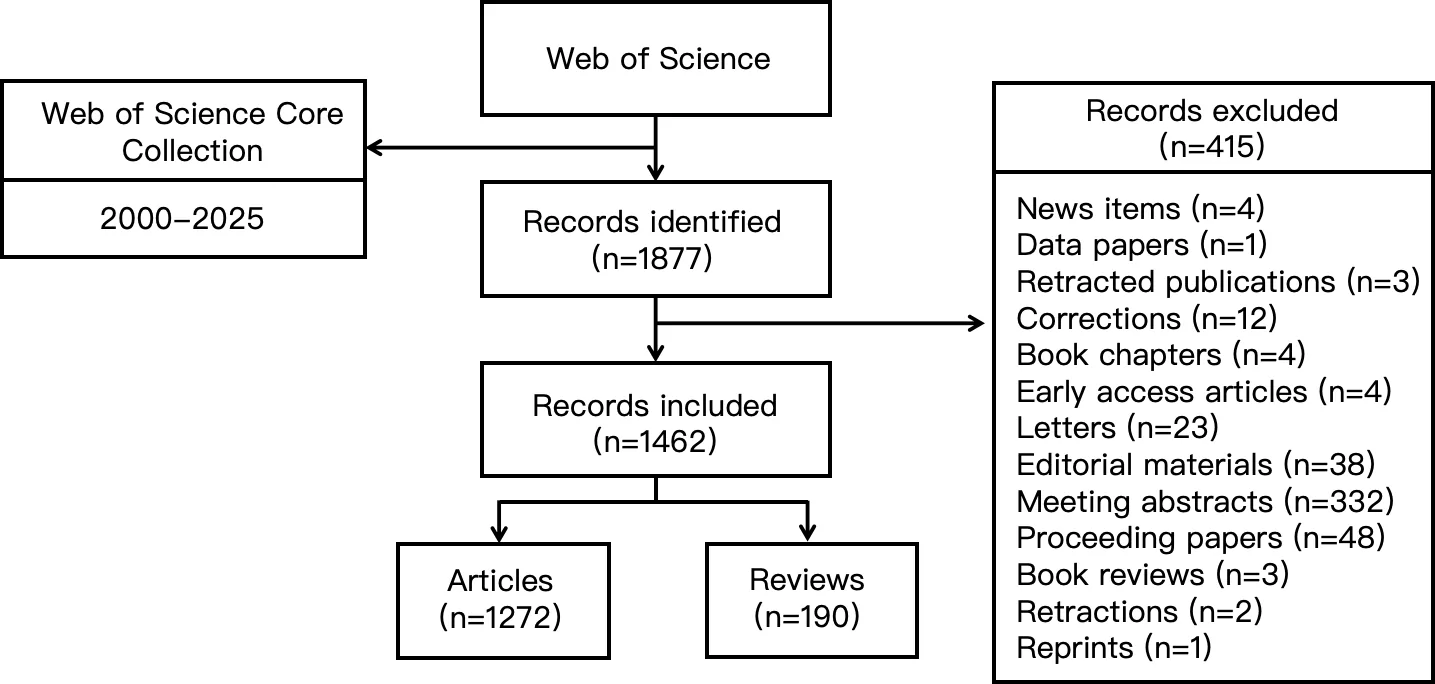
Flowchart for identifying and selecting publications. The document-type categories listed are not mutually exclusive, because some records may be assigned to multiple document types.

The complete data collection and filtering process is illustrated in Figure 1.

### Overview of Research Trends

**Figure 2.**
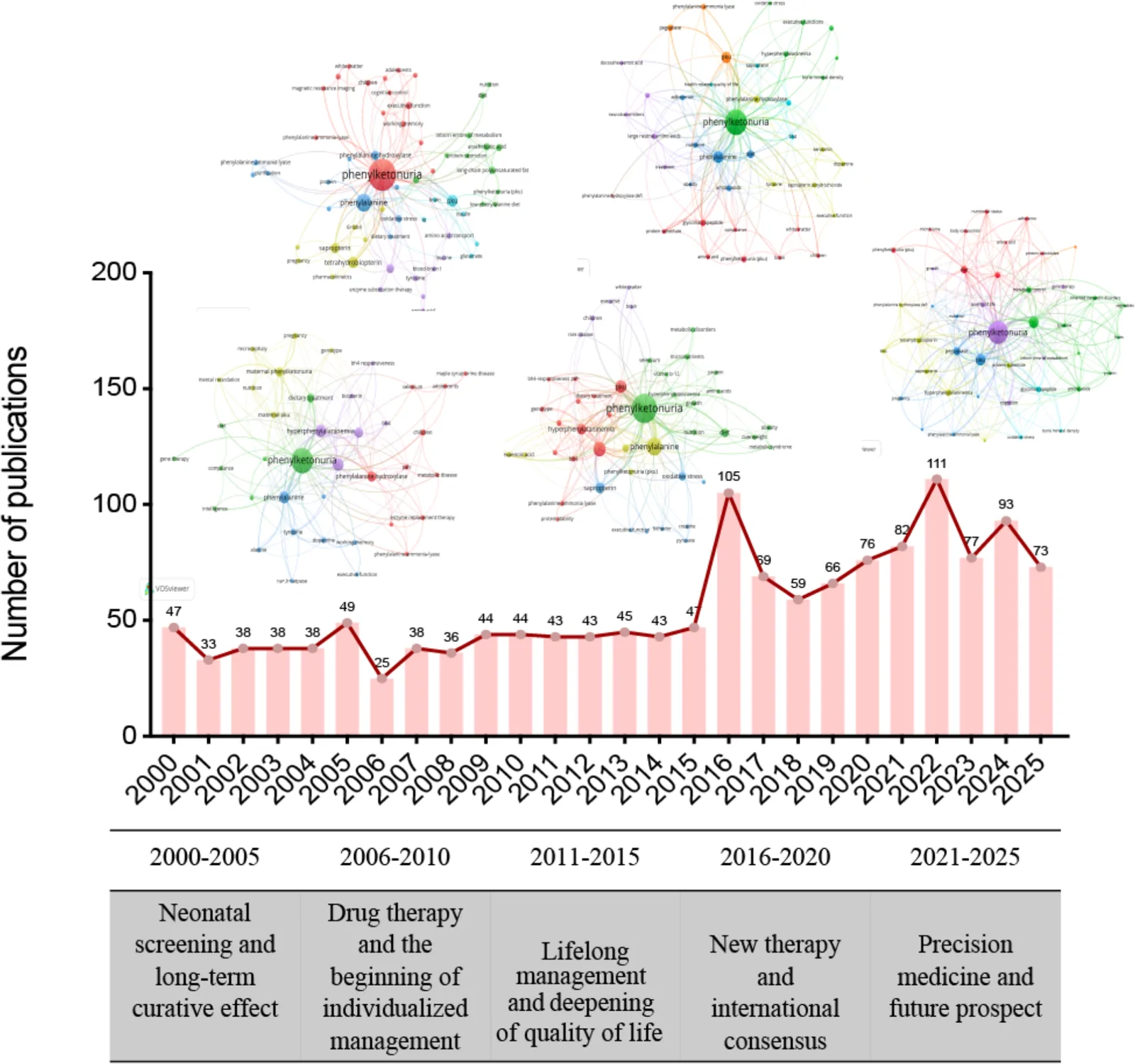
Annual publication volume and 5-year keyword cluster evolution analysis.

The temporal distribution of publications provides a clear depiction of research progress in the field of PKU. As illustrated in Figure 2, annual publication output remained relatively stable from 2000 to 2015, with a mean of 40.63 (SD 5.86) publications per year. A modest peak was observed in 2005 (49 publications), followed by a brief decline in 2006 (25 publications). The increase in publication activity coincided with growing interest in sapropterin-based therapies. Although publication numbers fluctuated somewhat in subsequent years, they consistently remained above pre-2015 levels, culminating in a peak of 111 articles in 2022. Despite a slight decline after 2022, annual output demonstrated only minor variation, reflecting sustained scholarly interest and ongoing innovation in this field.

In addition to publication trends, Figure 2 presents a comparative keyword analysis using 5-year intervals beginning in 2000, capturing the evolution of research priorities and emerging thematic trends within the field. Keywords were ranked according to both frequency and centrality, with the top 30 keywords for each interval summarized in Table 1. This longitudinal analysis reveals a clear shift in research emphasis: from an early focus on preventing intellectual disability toward improving long-term quality of life, and from managing metabolic abnormalities to developing potentially curative therapeutic strategies, and, more recently, toward emerging approaches such as gene therapy aimed at providing sustained metabolic correction [9,10].

**Table 1.** Top 30 keywords across 5-year intervals.

Rank	2000‐2005		2006-2010		2011-2015		2016-2020		2021-2025	
	Keywords	Occurrences, n	Keywords	Occurrences, n	Keywords	Occurrences, n	Keywords	Occurrences, n	Keywords	Occurrences, n
1	Phenylketonuria	89	Phenylketonuria	78	Phenylketonuria	125	Phenylketonuria	150	Phenylketonuria	234
2	Hyperphenylalaninemia	22	Phenylalanine	24	Phenylalanine	47	Phenylalanine	34	PKU	58
3	PKU	22	PKU	11	Tetrahydrobiopterin	30	PKU	30	Phenylalanine	57
4	Phenylalanine	19	Tetrahydrobiopterin	10	PKU	24	Diet	15	Pegvaliase	30
5	Phenylalanine; hydroxylase	14	Sapropterin	8	Sapropterin	18	Phenylalanine hydroxylase	12	Diet	25
6	Tetrahydrobiopterin	14	Phenylalanine hydroxylase	7	Hyperphenylalaninemia	16	Glycomacropeptide	11	Hyperphenylalaninemia	17
7	Maternal phenylketonuria	12	Hyperphenylalaninemia	6	Diet	11	Pegvaliase	10	Inborn errors of metabolism	17
8	Dietary treatment	11	Arachidonic acid	4	Oxidative stress	10	Amino acids	9	Glycomacropeptide	16
9	Children	6	Docosahexaenoic acid	4	Tyrosine	9	Phenylketonuria (PKU)	9	Metabolic control	16
10	Maternal PKU	6	Executive function	4	Phenylketonuria (PKU)	6	Hyperphenylalaninemia	8	Protein substitute	14
11	Tyrosine	6	Gene therapy	4	BH4	5	Nutrition	8	Phenylketonuria (pku)	13
12	Dopamine	5	Long-chain polyunsaturated fatty acids	4	Docosahexaenoic acid	5	Phenylalanine ammonia lyase	8	Tetrahydrobiopterin	13
13	Pregnancy	5	Oxidative stress	4	Growth	5	Tetrahydrobiopterin	8	Amino acids	12
14	Alanine	4	Amino acid transport	3	Micronutrients	5	Adherence	7	Sapropterin	11
15	Blood-brain barrier	4	Children	3	Phenylalanine hydroxylase	5	Executive functions	7	Cognition	10
16	Enzyme replacement therapy	4	Diet	3	Children	4	Inborn error of metabolism	7	Quality of life	10
17	Microcephaly	4	Diffusion tensor imaging	3	Genotype	4	Large neutral amino acids	7	Adherence	9
18	Nutrition	4	Inborn errors of metabolism	3	Inborn error of metabolism	4	Quality of life	7	Pregnancy	9
19	PAH	4	Phenylalanine ammonia lyase	3	Inborn errors of metabolism	4	Amino acid	6	Tyrosine	9
20	Working memory	4	Phenylketonurias	3	Metabolic disorders	4	Dopamine	6	Inherited metabolic disorders	8
21	Adolescents	3	Tyrosine	3	Nutrition	4	Sapropterin dihydrochloride	6	Nutritional status	8
22	BH4	3	Working memory	3	Obesity	4	Tyrosine	6	Oxidative stress	8
23	BH4 responsiveness	3	6R-BH4	2	Overweight	4	BH4	5	BH4	7
24	Biopterin	3	Activated carbon	2	Phenylalanine ammonia lyase	4	Bone mineral density	5	Body composition	7
25	Compliance	3	Adeno-associated virus	2	Quality of life	4	Children	5	Gene therapy	7
26	Diet	3	Adolescents	2	Sapropterin dihydrochloride	4	Docosahexaenoic acid	5	Phenylalanine hydroxylase deficiency	7
27	Executive function	3	Amino acid	2	Selenium	4	Protein substitute	5	Bone mineral density	6
28	Gene therapy	3	Blood-brain barrier	2	Amino acids	3	Sapropterin	5	Inborn error of metabolism	6
29	Genotype	3	Brain	2	Behavior	3	Serotonin	5	Nutrition	6
30	Inborn errors of metabolism	3	Cell transplantation	2	BH4-responsiveness	3	Brain	4	Phenylalanine ammonia lyase	6

### Country Analysis

**Figure 3.**
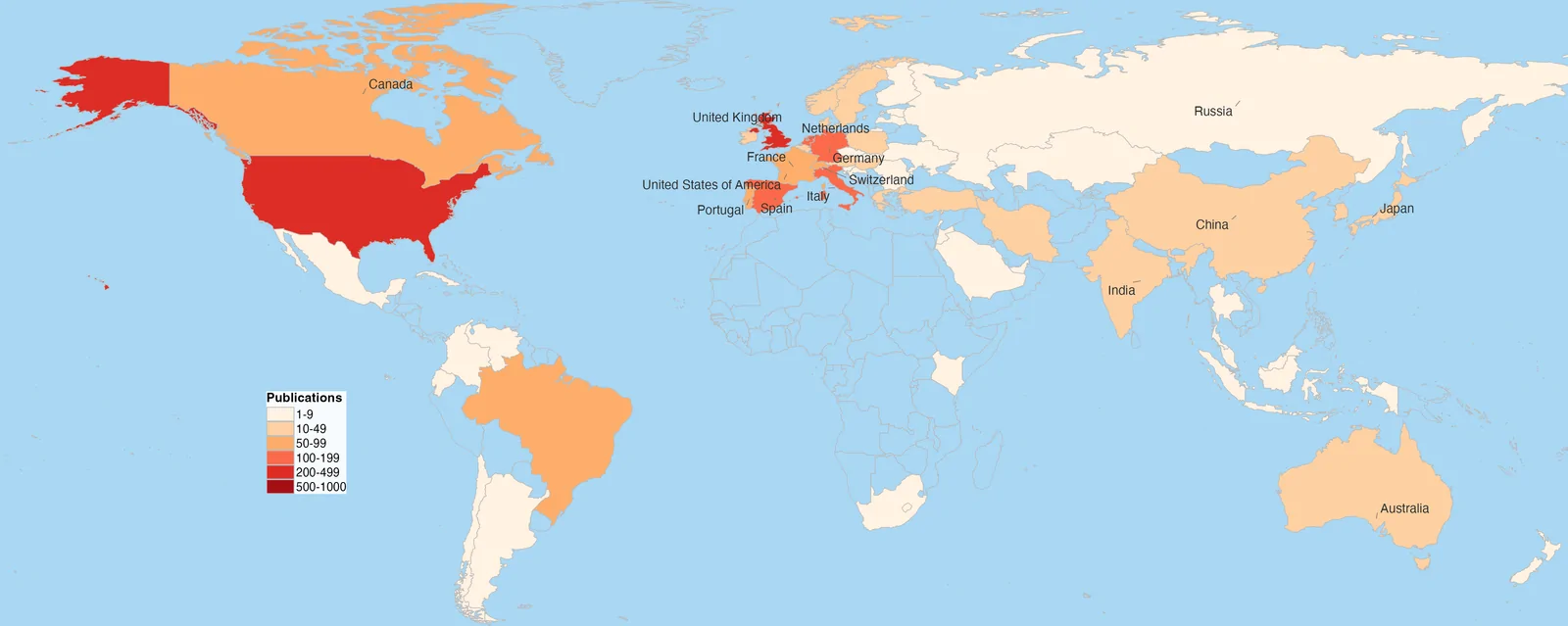
Distribution of publications.

A total of 64 countries contributed to the global publication output on PKU. As shown in Table 2, the top 10 productive countries were ranked based on their scientific output. The United States dominated the field with 375 publications and 11,142 citations, confirming its central role in both research productivity and scholarly influence. The United Kingdom ranked second in publication volume (185 publications) and citation impact (4269 citations), and also demonstrated the highest level of international collaboration, reflecting its strong integration within the global scientific community. The worldwide distribution of publications is depicted in Figure 3.

**Table 2.** Top 10 countries in terms of publication output.

Rank	Country	Documents, n	Citations, n	Total link strength, n
1	United States	375	11,142	188
2	United Kingdom	185	4269	274
3	Germany	144	4004	247
4	Italy	121	2806	182
5	The Netherlands	103	3281	199
6	Spain	85	2140	169
7	Switzerland	72	2560	105
8	China	67	2174	21
9	Canada	58	1931	64
10	Australia	35	1719	16

Given the higher prevalence of PKU among Caucasian populations, European countries exhibited strong representation in the literature. Germany, the Netherlands, Italy, and Switzerland ranked third through sixth, with publication counts of 144, 103, 121, and 72 publications, and 4004, 3281, 2806, and 2560 citations, respectively. Notably, Germany displayed the second-highest collaboration intensity, which exceeded even that of the United States.

**Figure 4.**
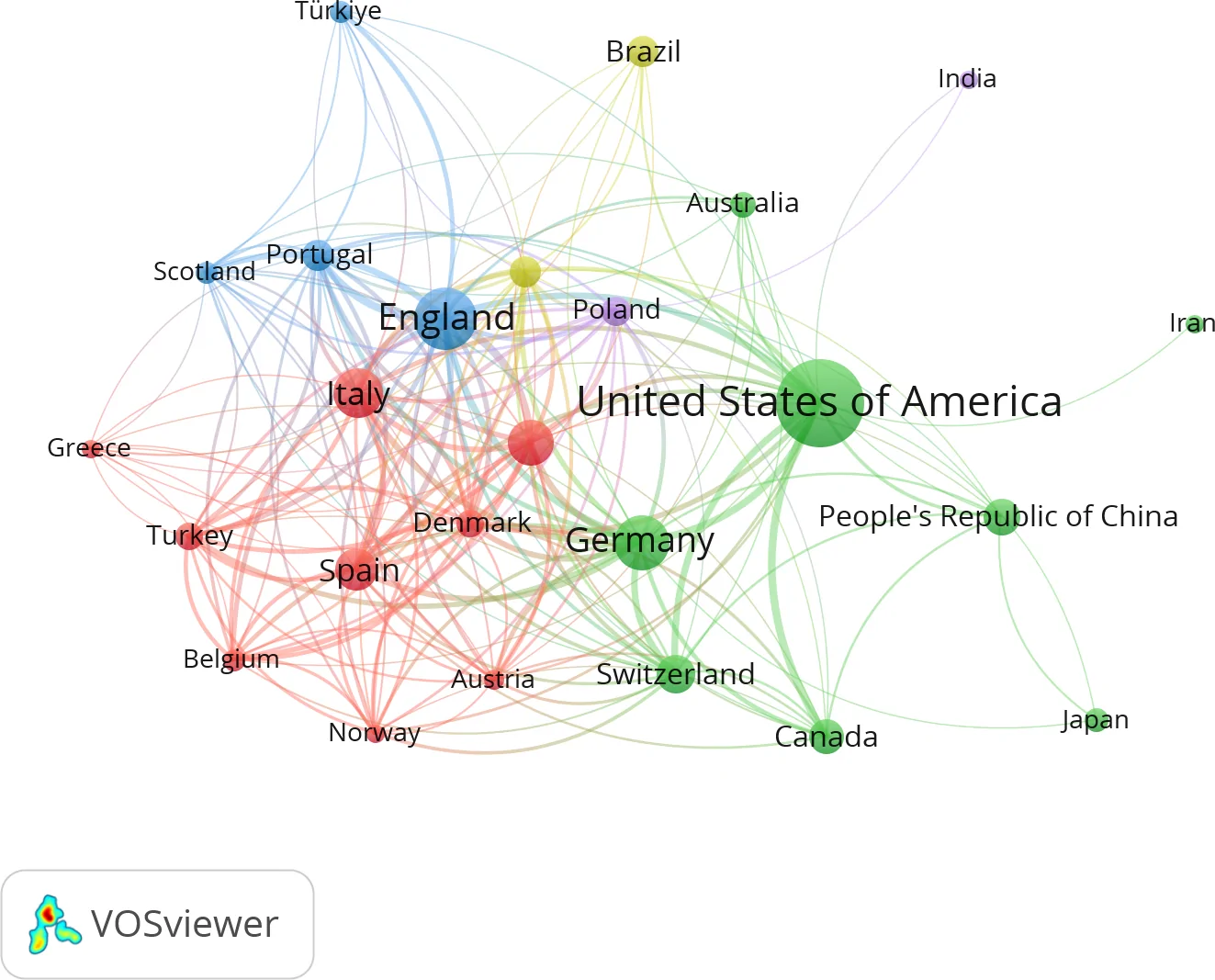
Map of the national cooperation network.

**Figure 5.**
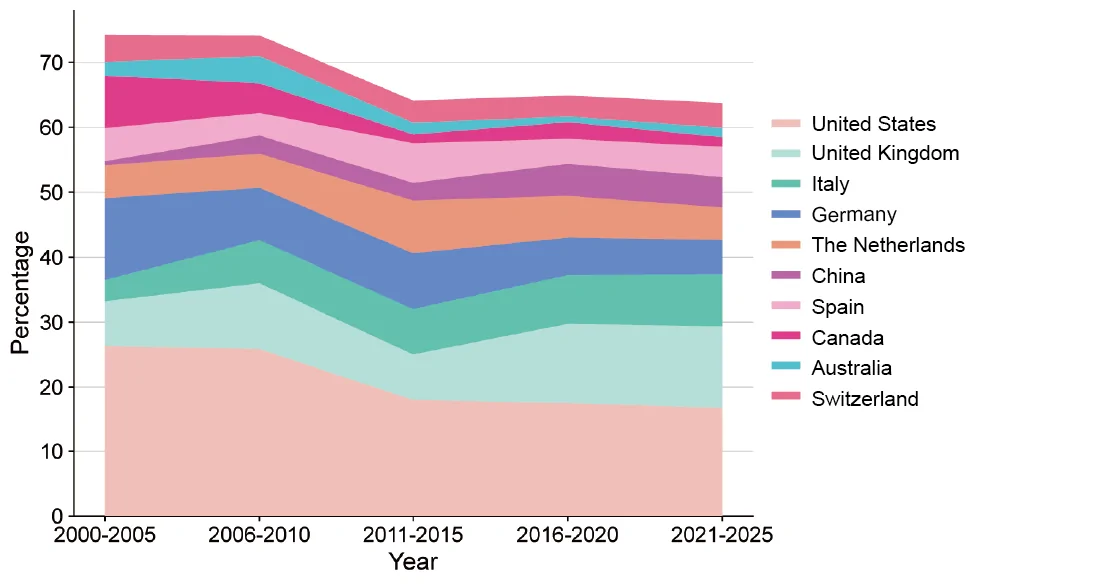
Percentage of publications by country, displayed in 5-year intervals over the 25-year period.

Annual publication trends for these 10 leading countries are illustrated in Figure 4. VOSviewer was used to construct a geographical visualization of national contributions and collaborative patterns, applying a minimum threshold of 5 publications per country. This criterion yielded 38 countries included in the network analysis. In the resulting visualization, node size corresponds to publication output, whereas link thickness corresponds to the strength of collaborative relationships. Additionally, Figure 5 depicts the temporal evolution of the top 10 countries’ publication shares as a percentage of total global output, displayed in 5-year intervals over the 25-year period. The United States consistently maintained the largest share of publications, although its relative proportion declined slightly over time. In contrast, the United Kingdom and Italy exhibited a moderate upward trend in their publication shares.

### Author Analysis

**Figure 6.**
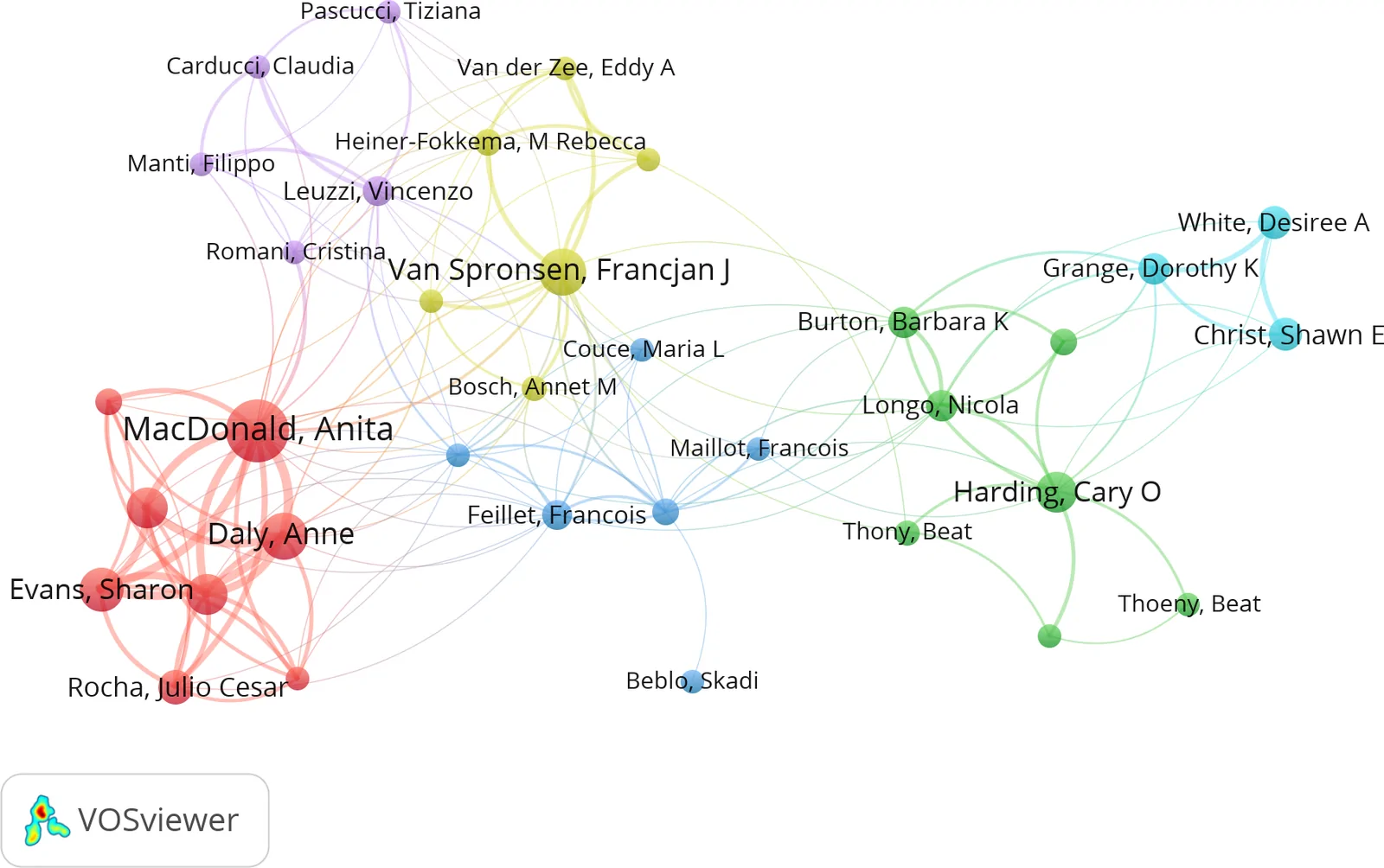
Author collaboration network.

A total of 5359 researchers contributed to PKU-related publications. Figure 6 presents the author collaboration network, visualizing the collaborative relationships among the most productive and influential researchers in this field. As shown in Table 3, Anita MacDonald from the United Kingdom lead the field with 92 publications and 1613 citations. Their substantial involvement in developing international diagnostic and treatment guidelines, widely regarded as the “gold standard” for PKU management, has resulted in a particularly high citation count [11]. Similarly, Francjan J van Spronsen from the Netherlands, a major contributor to international consensus guidelines, ranks second with 40 publications and 1058 citations, demonstrating extensive collaboration with MacDonald [11,12].

**Table 3.** Top 10 authors in terms of publications.

Rank	Author (surname, forename)	Documents, n	Citations, n	Total link strength, n
1	MacDonald, Anita	92	1613	182
2	Van Spronsen, Francjan J	40	1058	64
3	Daly, Anne	36	272	143
4	Harding, Cary O	32	1009	41
5	Evans, Sharon	31	254	132
6	Ashmore, Catherine	28	149	120
7	Pinto, Alex	28	191	123
8	Christ, Shawn E	20	486	24
9	White, Desiree A	20	514	30
10	Burton, Barbara K	19	543	33

Beyond these 2 international leaders, several additional researchers from the United Kingdom, including Anne Daly, Sharon Evans, and Catherine Ashmore, form the core of European research on PKU clinical management. In parallel, US investigators, including Cary O Harding, Shawn E Christ, Desiree A White, and Barbara K Burton, have played key roles in translating foundational scientific discoveries into innovative therapies that have contributed substantially to the development and clinical evaluation of treatments later approved by regulatory agencies, thereby establishing the US central position in PKU research.

### Institutional Analysis

**Figure 7.**
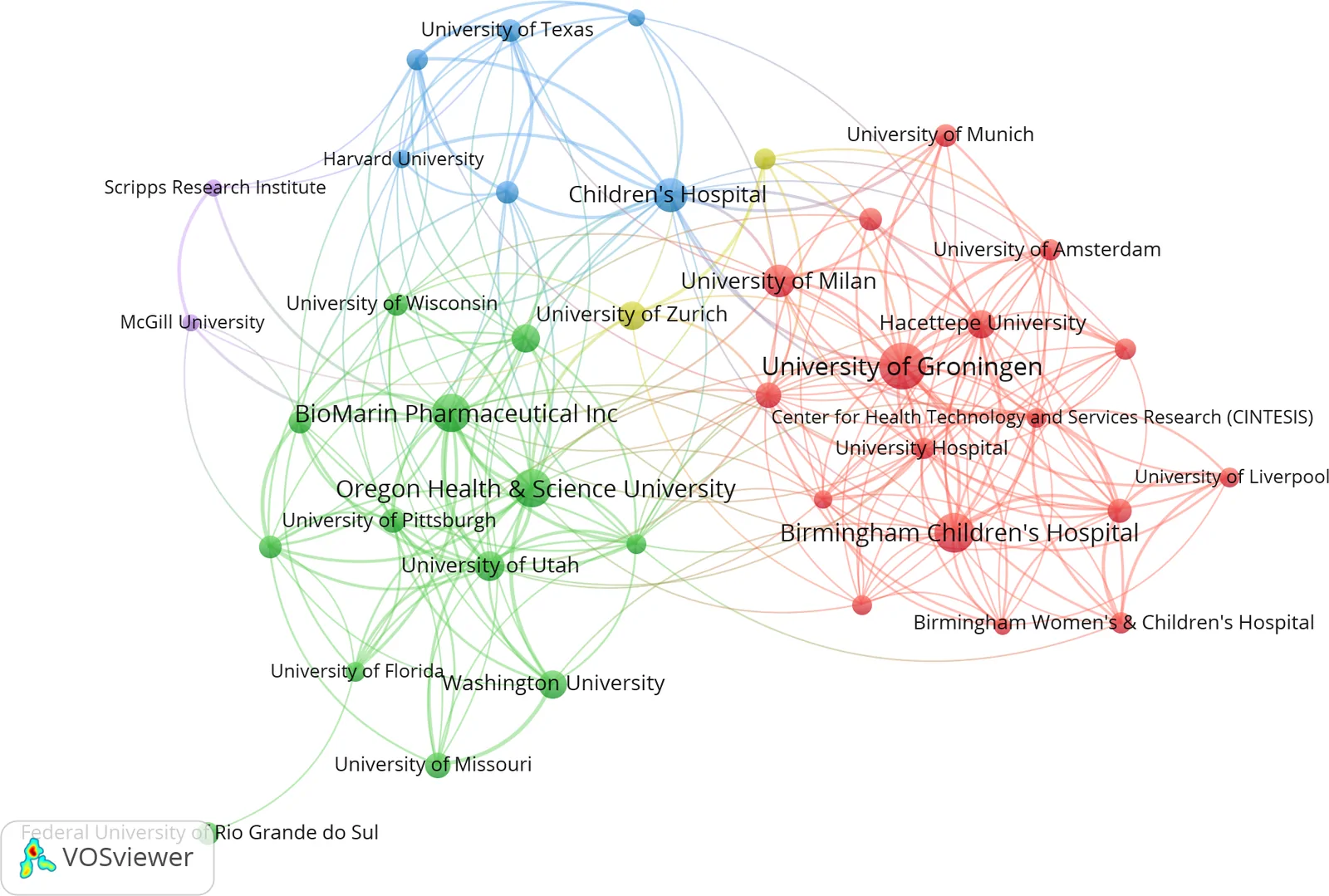
Network map of cooperation among organizations.

A total of 1708 institutions contributed to PKU-related publications. Using a minimum threshold of 13 publications per institution, VOSviewer generated a collaborative network composed of 41 institutional nodes. Figure 7 presents the resulting network map of cooperation among organizations, visualizing the collaborative relationships and clustering patterns of the most productive institutions in this field. Table 4 lists the top 10 institutions ranked by publication volume. Table 4 lists the top 10 institutions ranked by publication volume. The University of Groningen led with 68 publications and 2006 citations and exhibited the highest degree of collaborative connectivity. As PKU is an inherited metabolic disorder frequently managed in pediatric settings, children’s hospitals have played an important role in advancing the field. Birmingham Children’s Hospital ranked second with 52 publications and 1242 citations.

**Table 4.** Top 10 institutions by publication output.

Rank	Organization	Documents, n	Citations, n	Total link strength, n
1	University of Groningen	68	2006	102
2	Birmingham Children’s Hospital	52	1242	49
3	Oregon Health & Science University	50	1546	89
4	Biomarin Pharmaceutical Inc	48	1548	96
5	Children’s Hospital	40	1285	79
6	University of Milan	36	844	31
7	The University of Utah	32	1230	78
8	Hacettepe University	30	558	62
9	University of Zurich	29	1191	24
10	Washington University	29	1036	46

Overall, institutions active in PKU research maintain strong and extensive collaborative relationships, forming a highly cohesive scientific community. The dense interinstitutional linkages observed in the network map indicate a well-integrated research ecosystem that facilitates efficient knowledge exchange and the rapid translation of scientific discoveries into clinical practice.

### Journal Analysis

**Figure 8.**
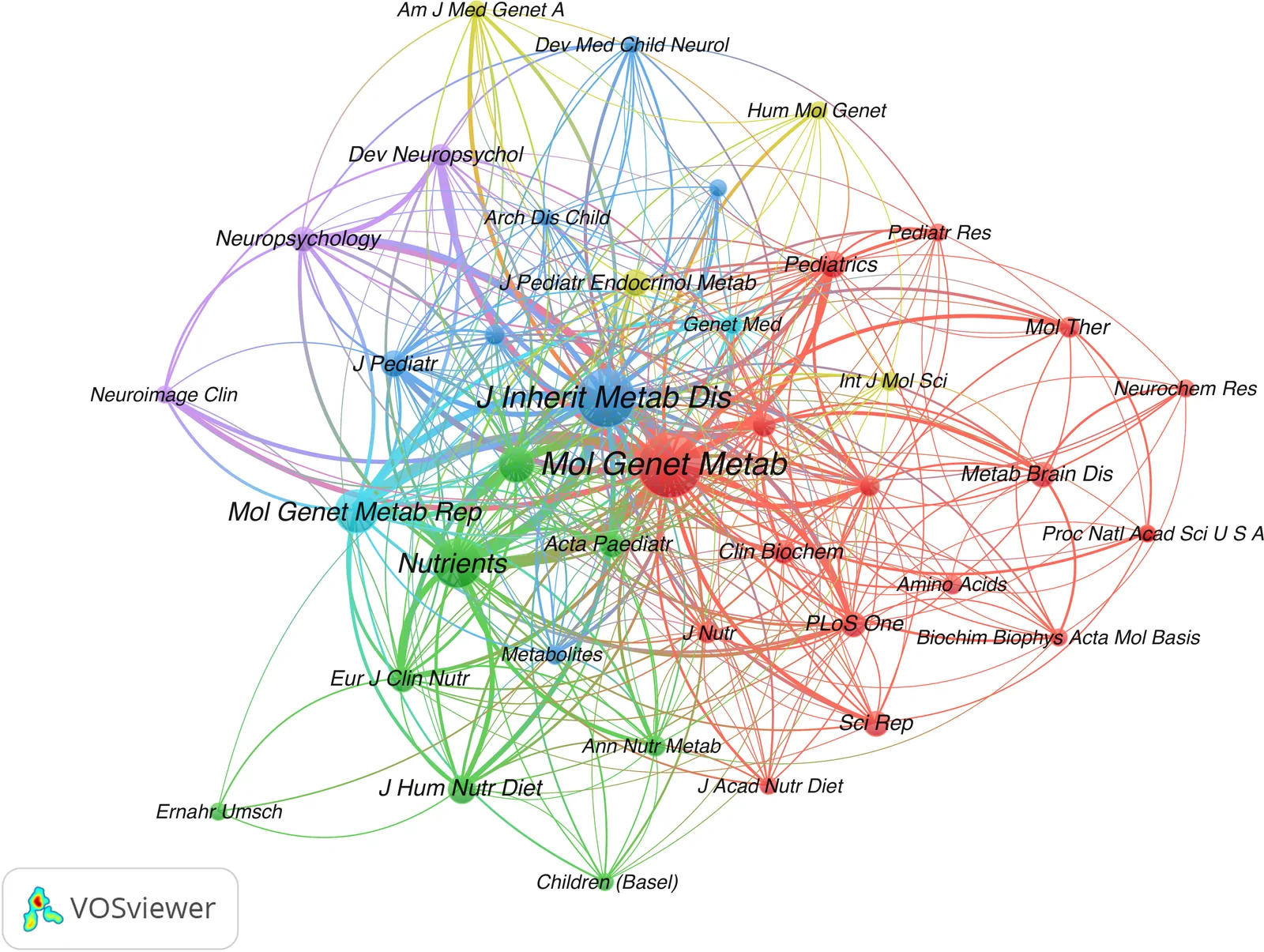
Journal co-citation network.

Journal analysis identified the core academic domains contributing to PKU research. The publications were distributed across 429 journals, of which 40 published at least 5 PKU-related articles. Figure 8 presents the journal co-citation network, visualizing the intellectual structure and disciplinary clustering of journals that have significantly contributed to PKU research. Table 5 summarizes the top 10 journals, including their Journal Citation Reports category, impact factor (IF), number of articles, total citations, publisher, and country of origin. *Molecular Genetics and Metabolism* ranked first in both the number of articles and total citations, reflecting its central role in disseminating research directly related to the genetic and metabolic aspects of PKU. Most high-frequency journals were associated with genetics, metabolism, pediatrics, nutrition, rare diseases, and endocrinology.

Although journals with moderate impact factors account for the majority of publications, several high-impact international journals have also featured PKU-related research. *The Lancet* (IF=98.4), *The New England Journal of Medicine* (IF=74.7), *Nature Medicine* (IF=58.7), *Cell Host & Microbe* (IF=30.3), and *Nature Communications* (IF=16.6) have each published 1 to 2 articles on PKU. Despite the small output, these publications exhibit citation frequencies far above the field average, highlighting both the visibility of PKU research and the importance of its broader clinical and scientific implications.

**Table 5.** Top 10 journals in publication.

Rank	Journal name	2023 IF^a^	JCR^b^ category rank (quartile)	Publisher and country	Documents, n	Citations, n	Total link strength, n
1	*Molecular Genetics and Metabolism*	2.9	Q3 (Biochemistry & Molecular Biology)	Elsevier, United States	151	4506	1259
2	*Journal of Inherited Metabolic Disease*	4.6	Q2 (Genetics & Heredity)	Wiley, United States	116	3612	911
3	*Nutrients*	5.9	Q1 (Nutrition & Dietetics)	MDPI, Switzerland	76	570	620
4	*Molecular Genetics and Metabolism Reports*	1.9	Q4 (Biochemistry & Molecular Biology)	Elsevier, United States	56	759	536
5	*Orphanet Journal of Rare Diseases*	3.7	Q2 (Medicine, Research & Experimental)	Springer Nature, United Kingdom	32	560	334
6	*Journal of Human Nutrition and Dietetics*	3.3	Q2 (Nutrition & Dietetics)	Wiley, United Kingdom	17	273	115
7	*Journal of Pediatrics*	3.3	Q2 (Pediatrics)	Elsevier, United States	15	523	160
8	*Pediatrics*	5.8	Q1 (Pediatrics)	American Academy of Pediatrics, United States	15	470	123
9	*Journal of Pediatric Endocrinology & Metabolism*	1.8	Q4 (Pediatrics) or Q4 (Endocrinology & Metabolism)	Walter de Gruyter, Germany	14	91	60
10	*PLOS One*	3.2	Q2 (Multidisciplinary Sciences)	PLOS, United States	13	319	128

a IF: impact factor.

b JCR: Journal Citation Reports.

### Keyword Analysis

**Figure 9.**
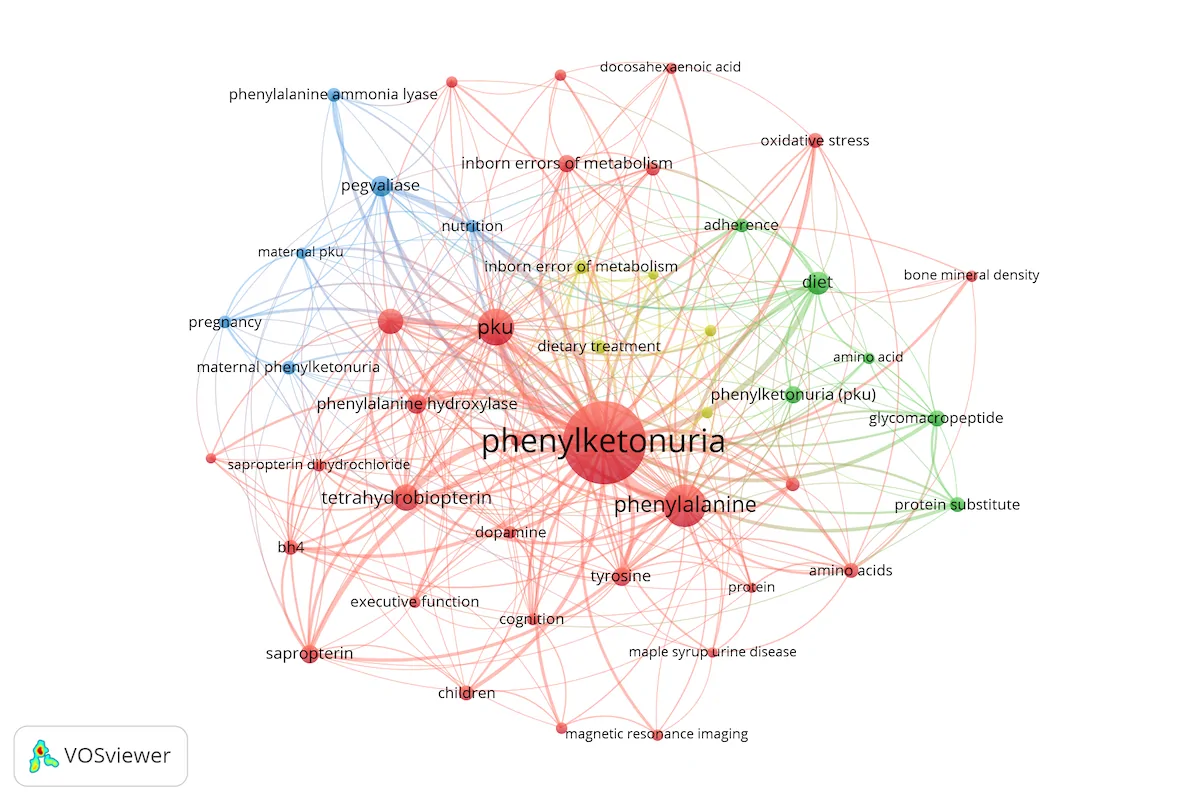
Keyword co-occurrence network.

A total of 2291 keywords were extracted from the included publications. Among these, 46 terms meeting the threshold of ≥10 occurrences were selected for co-occurrence network analysis. Figure 9 presents the resulting keyword co-occurrence network. In the visualization, node size represents keyword frequency, color gradient indicates the temporal distribution of research attention (cool colors for earlier periods, warm colors for recent hotspots), and edge thickness reflects co-occurrence strength.

The results identified “phenylketonuria” (576 occurrences, centrality =0.74) as the dominant core term, forming a tightly connected research cluster with “phenylalanine metabolism” (156 occurrences), “tetrahydrobiopterin therapy” (60 occurrences), and “enzyme replacement therapy” (38 occurrences). In the domain of nutritional management, “dietary intervention” (46 occurrences), together with “protein substitutes” (19 occurrences) and “glycomacropeptide application” (23 occurrences), constituted the principal research axis. The strong association between “genotype analysis” (10 occurrences) and “hyperphenylalaninemia” (56 occurrences) highlights the increasing integration of precision medicine concepts into clinical practice. Notably, although terms such as “neurocognitive function” (13 occurrences) and “white matter integrity” (11 occurrences) appeared with moderate frequency, their relatively high betweenness centrality values (0.16‐0.19) indicate that they act as critical bridging nodes, connecting biochemical research with clinical outcome studies and signaling their importance as emerging research foci.

Temporal evolution analysis revealed 3 distinct phases of shifting research priorities. In the early phase, between 2000 and 2009, research predominantly centered on “classic dietary therapy” and “newborn screening system optimization.” In the second phase, from 2010 to 2017, research transitioned toward precision medicine directions, including “tetrahydrobiopterin responsiveness” and “genotype-phenotype correlations.” The most recent phase, from 2018 to 2024, converged on 3 cutting-edge directions: “pegylated enzyme preparations” (burst strength 7.86), “metabolomics analysis” (strength 6.24), and “oxidative stress monitoring” (strength 5.93). These areas reflect a paradigm shift toward molecularly targeted therapies and dynamic metabolic monitoring approaches.

**Figure 10.**
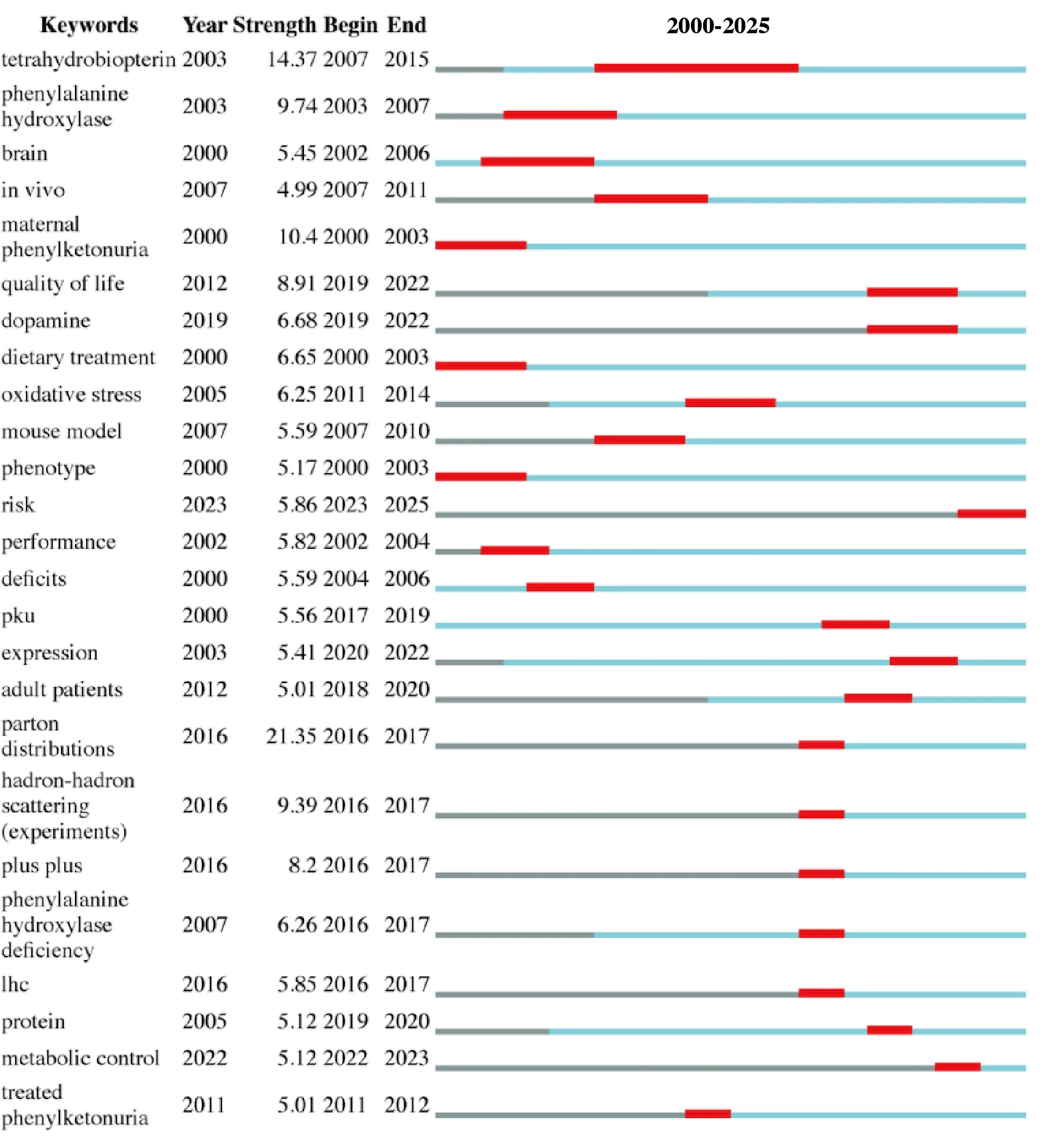
Top 25 keywords with the strongest citation bursts. The colors represent clusters automatically generated by VOSviewer. LHC: Large Hadron Collider.

Figure 10 illustrates the top 25 keywords with the strongest citation bursts, which further demonstrate 3 distinct evolutionary phases. Analysis of these top 25 keywords with the strongest citation bursts further demonstrates 3 distinct evolutionary phases. The early phase (2000‐2007) was defined by breakthroughs in biochemical mechanisms and cofactor therapy, as indicated by strong bursts for “tetrahydrobiopterin” (14.37) and “phenylalanine hydroxylase,” marking breakthroughs in cofactor therapy and metabolic pathway research. Concurrent bursts for “maternal PKU” (10.4) and “dietary treatment” highlighted foundational work in clinical management. The second phase (2011‐2020) witnessed a shift toward neurocognitive and long-term outcome research. During this period, “oxidative stress” (6.25) revealed novel pathological mechanisms, while emerging terms such as “quality of life” (8.91) and “adult patients” marked the growing emphasis on holistic and lifespan-oriented management. The recent phase (2022‐2025) exhibits precision management trends, with the keyword burst for “metabolic control” (5.12) highlighting increasing attention to individualized therapy, therapeutic optimization, and comprehensive outcome evaluation. Collectively, these trends reflect an overarching transition in PKU research from mechanistic exploration to clinical refinement, and ultimately toward precision and personalized medicine.

### Reference Co-Citation Analysis

**Figure 11.**
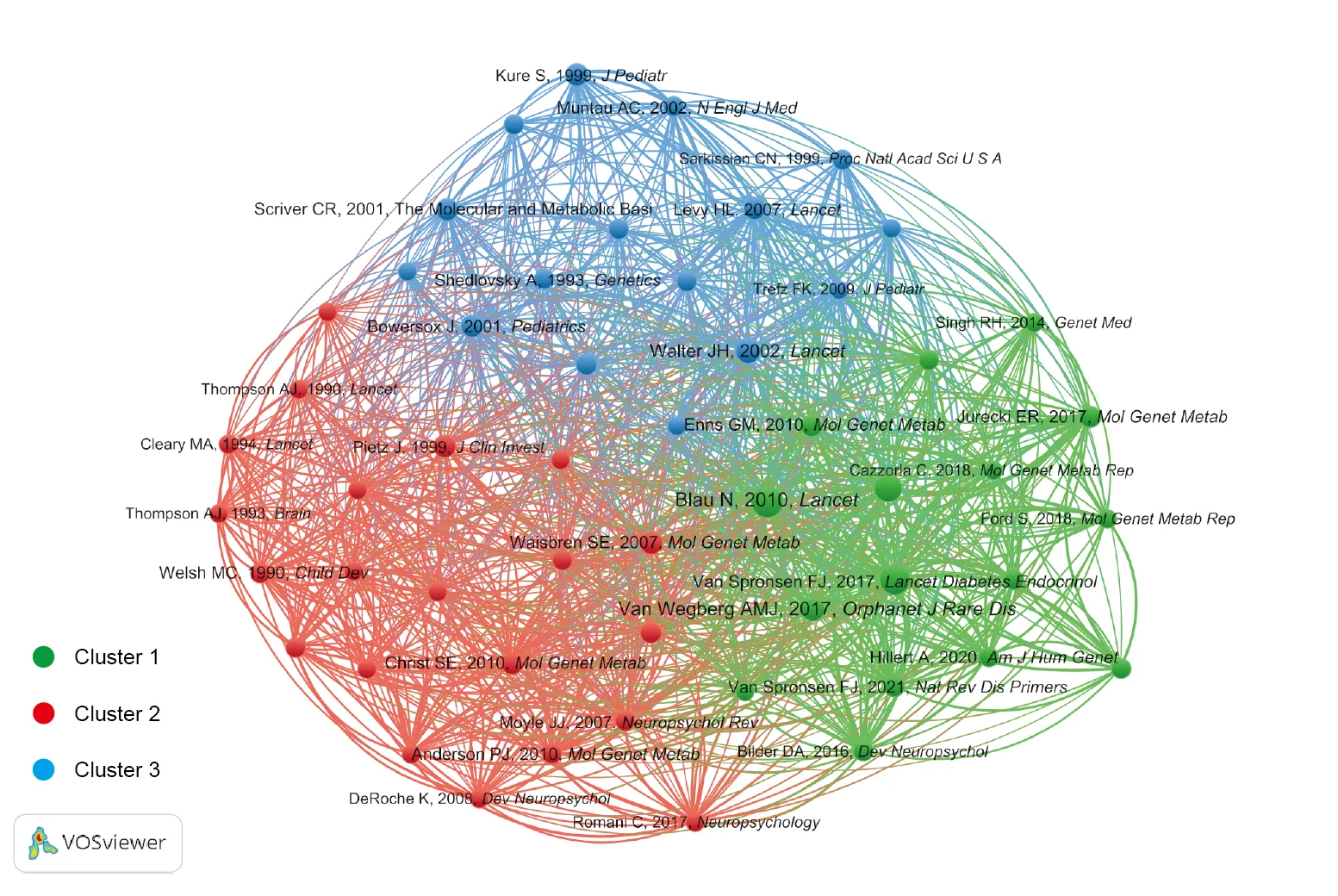
Co-citation of references [1,2,11-39]. The colors represent different clusters automatically generated by VOSviewer based on the co-occurrence analysis.

**Figure 12.**
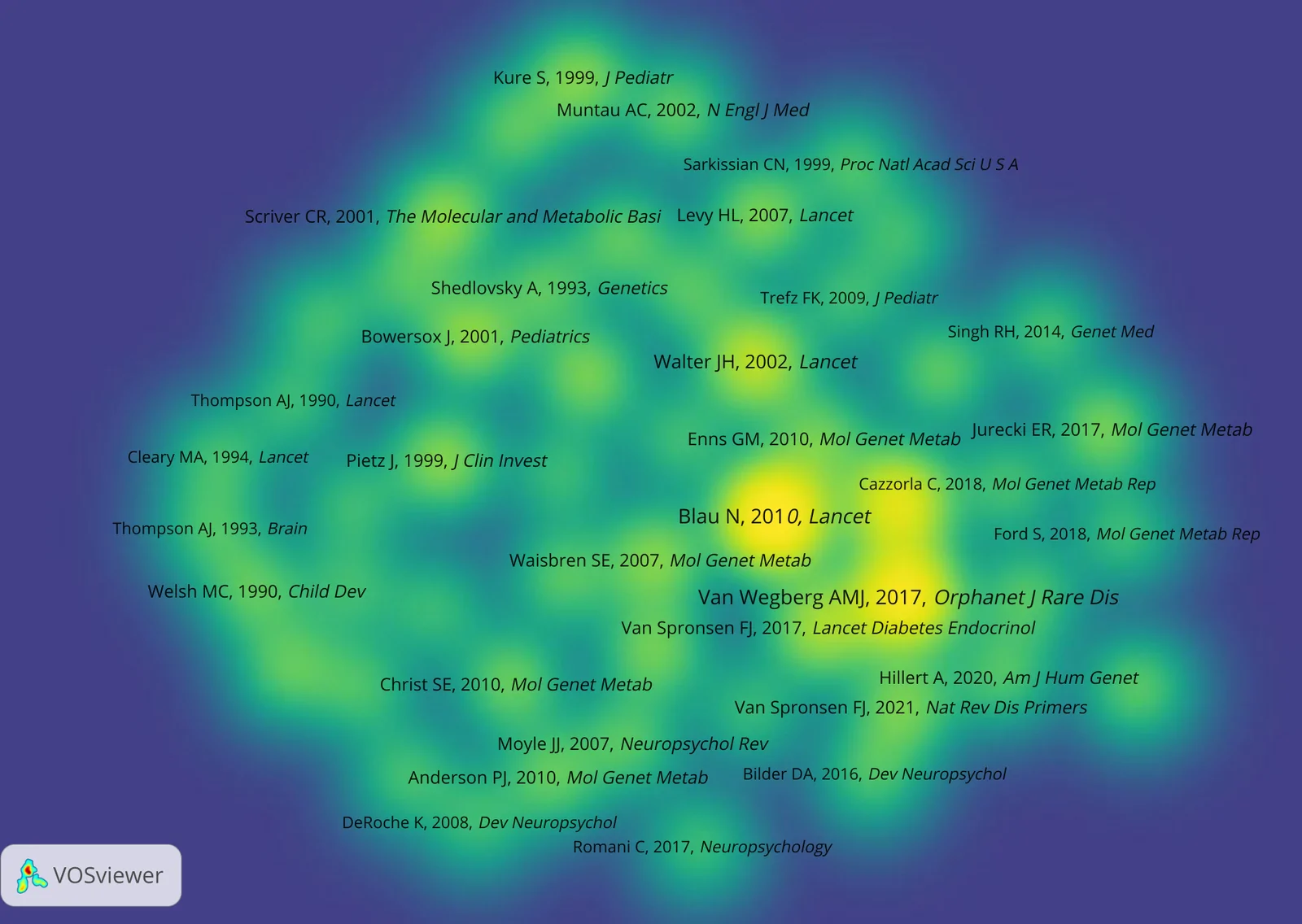
Hotspot map of co-cited references [1,2,11-39].

Figure 11 displays the reference co-citation network of foundational literature in PKU research, with node size reflecting citation frequency and edge thickness denoting co-citation strength. The structure reveals several distinct yet interrelated knowledge domains that collectively constitute the intellectual foundation of PKU research. Additionally, Figure 12 presents the same co-citation network in the form of a heatmap, providing an alternative visualization of node importance and clustering intensity.

The most prominent node in the network is Blau et al [1] (*Lancet*, cited 233 times), which functions as a central integrative hub. This landmark publication on sapropterin responsiveness demonstrates strong co-citation links with both clinical management literature [11,12] and basic biochemical studies [17,19,40]. Its position indicates its significant role in bridging mechanistic insights with therapeutic applications.

Two major intellectual lineages emerge clearly from the network: one lineage, the clinical management tradition, connects early seminal works on PKU diagnosis and treatment [15,41] (with later authoritative guidelines and consensus statements [20]. Meanwhile, a second lineage, the basic science and mechanistic tradition, links foundational biochemical studies [17,42,43] with recent therapeutic advances [2].

The network also highlights strong co-citation clusters connecting neurocognitive outcome studies [21,22,44,45] with metabolic research [16,46]. This relationship reflects the long-standing scientific recognition that metabolic control is intimately linked to neurological integrity and cognitive development in PKU. Notably, historical milestones [23,47] maintain enduring co-citation ties with contemporary research, emphasizing the cumulative and evolving nature of knowledge construction in this field.

Overall, the co-citation patterns demonstrate a highly integrated research ecosystem, characterized by dynamic interplay between basic science discoveries and clinical innovation. This close coupling between bench and bedside has enabled rapid translation of mechanistic findings into therapeutic advances (Table 6).

**Table 6.** Top 10 most cited articles in publications.

Rank	Cited reference	Citations (n)	Total link strength (n)
1	Blau et al [1], 2010	233	884
2	van Wegberg et al [11], 2017	192	788
3	Vockley et al [48], 2014	150	746
4	Walter et al [15], 2002	112	480
5	van Spronsen et al [12], 2017	94	413
6	Bowersox [24], 2001	80	330
7	Pietz et al [16], 1999	78	287
8	Scriver and Kaufman [17], 2001	78	216
9	Waisbren et al [25], 2007	76	400
10	Kure et al [14], 1999	74	226

## Discussion

### Principal Findings

This bibliometric analysis provides a comprehensive overview of global PKU treatment research from 2000 to 2025. The findings reveal a sustained increase in publication output over the past 25 years, reflecting growing scientific and clinical interest in PKU management. The United States and several European countries remain the dominant contributors to the field, supported by extensive international collaboration networks. Keyword co-occurrence, citation burst, and thematic evolution analyses demonstrate a gradual transition from traditional dietary management toward pharmacological interventions, enzyme substitution therapies, neurocognitive outcome assessment, and precision medicine–oriented approaches. Collectively, these findings highlight the increasing diversification of PKU research and suggest that future investigations are likely to focus on individualized therapeutic strategies and emerging disease-modifying technologies.

### General Information

Since 2000, global research on PKU has advanced substantially. The United States and several European countries, particularly the Netherlands, Germany, and the United Kingdom, have formed the central research axis in this field. These countries exhibit not only high publication productivity but also strong citation performance, reflecting their long-standing research foundations and academic leadership [1,11,12,48-51]. The current scientific landscape is distinctly internationalized, with dense collaborative links between North American and European institutions, as well as strong intra-European academic networks.

At the institutional and researcher levels, major contributions have largely originated from specialized metabolic centers and long-established clinical research groups. In the United States, researchers such as Cary O. Harding have been highly influential contributors to research on gene therapy and on pharmacological interventions in PKU and pharmacological innovation, with findings frequently published in high-impact journals, including *Molecular Genetics and Metabolism*, *The Lancet*, and *The American Journal of Clinical Nutrition* [52,53]. In the Netherlands, Francjan J. van Spronsen and Anita MacDonald have made seminal contributions in evidence-based dietary management, treatment optimization, and long-term outcome assessment, forming the empirical backbone for international PKU management guidelines [12,54]. Research teams in the United Kingdom further strengthened Europe’s leadership through influential work on neurocognitive development, metabolic phenotyping, and patient-centered outcome evaluation.

Large-scale multinational collaborations, most notably the European PKU Guidelines Working Group, as well as other long-term outcome research consortia, have generated landmark findings that clearly depict the relationship between early metabolic control and neurodevelopmental outcomes, paving the way for individualized treatment strategies [11]. The extensive and highly interconnected co-authorship networks observed in this study reflect these sustained collaborative efforts, highlighting not only the central roles of Europe and the United States but also the emerging contributions from Asia and Australia. Overall, these global research activities have deepened scientific understanding of PKU pathophysiology, mechanisms, and treatment strategies and also laid a solid foundation for advancing precision medicine and novel therapies for individuals living with PKU worldwide.

### Research Hotspots and Theme Evolution

Bibliometric analysis indicates that “metabolic control or nutritional management” constitutes the most central and persistently active research cluster in PKU, serving as the conceptual bridge linking major thematic domains over the past 2 decades. Converging evidence from both clinical and basic research supports a core consensus: achieving sustained and stable metabolic control is essential for optimizing long-term outcomes in patients with PKU [55]. Longitudinal cohort studies demonstrate that early-life fluctuations in blood phenylalanine levels are closely associated with later neurocognitive performance, while neuroimaging studies reveal that metabolic instability correlates strongly with disruptions in white matter microstructure [56]. Comprehensive systematic reviews, most notably the analysis by van Spronsen in *Molecular Genetics and Metabolism*, have validated a dose-response relationship between blood phenylalanine thresholds and neurological injury. Disturbances in metabolic homeostasis, particularly elevated phenylalanine to tyrosine ratios, impair neurotransmitter synthesis and myelination, thereby heightening vulnerability to neurodevelopmental impairment [57]. Furthermore, phenotype-genotype association studies have linked PAH mutation profiles with metabolic phenotypes and treatment responsiveness, and Blau et al [1] have advanced a genotype-guided framework for individualized therapeutic decision-making.

A second major keyword cluster focuses on “neurocognitive and behavioral outcomes,” encompassing concepts such as “executive function,” “white matter integrity,” and “cognitive development,” reflecting the ongoing investigation into the mechanisms of long-term neurological impairment in PKU. Tyrosine deficiency results in compromised prefrontal cortex function and reduced cognitive flexibility. These insights have broadened the research perspective from traditional metabolic monitoring to investigations of neurochemical pathways and functional brain networks, reframing PKU as a modifiable neurometabolic disorder. Consequently, the clinical priority has evolved from determining whether intellectual disability occurs to how to optimize neurodevelopmental pathways through early metabolic control [58].

The third prominent research direction centers on *therapeutic strategies* and *individualized interventions*. Keywords such as “tetrahydrobiopterin responsiveness,” “enzyme replacement therapy,” “large neutral amino acids,” and “glycomacropeptide” illustrate the transition from uniform dietary management to precision medicine [13,59-61]. Accumulating evidence supports the effectiveness of individualized therapeutic protocols tailored to genotype, metabolic phenotype, and pharmacological responsiveness, with demonstrated benefits for treatment adherence and quality of life. Current research priorities include defining optimal candidate populations for various treatment modalities (classic dietary therapy, cofactor treatment, and enzyme replacement therapy), developing models for predicting therapeutic efficacy, and refining protein substitute formulations to enhance metabolic stability. These research directions align with broader advancements in the management of inborn errors of metabolism and highlight the interdisciplinary integration of nutrition science, molecular biology, and pharmaceutical innovation [62,63].

From a chronological perspective, PKU research has progressed from macroscopic clinical observation to increasingly nuanced mechanistic understanding. During the early 2000s, investigations grounded in the “dietary control hypothesis” established treatment standards based on clinical observation and biochemical parameters. By approximately 2010, the rapid adoption of gene sequencing technologies propelled PAH genotype-phenotype correlations to the forefront, driving intensive examination of the metabolic consequences of specific mutations [64]. Since 2015, the emergence of long-term cohort studies, multimodal neuroimaging, and clinical trials of novel therapeutics has fostered the development of an integrated research paradigm that connects metabolic, genetic, neurocognitive, and behavioral dimensions of PKU [65,66]. Looking ahead, several areas hold strong potential for future breakthroughs: targeted modulation of key metabolic pathways to prevent neurotoxicity; integration of genetic backgrounds with metabolic phenotypes to identify high-risk subgroups and guide individualized interventions; and comprehensive characterization of the metabolic-brain-behavior axis in PKU through integrated models incorporating metabolomics, radiomics, and neurobehavioral profiling. Collectively, these advancements position precision metabolic management at the forefront of future PKU research and clinical practice [67,68].

### Influential Literature and Research Paradigm Transformation

The early theoretical foundation of PKU research was shaped by the “metabolic imbalance hypothesis,” which emerged in the late 20th century and was refined through the seminal work of Scriver [69]. Landmark cohort studies soon provided clinical validation for this hypothesis. Notably, investigations by Pietz et al [46] demonstrated a significant negative correlation between the duration of neonatal blood phenylalanine elevations above treatment thresholds and subsequent childhood IQ scores. These studies advanced the field beyond descriptive phenotypic characterization, establishing clear causal links between specific metabolic markers and neurodevelopmental outcomes, signaling a critical transition from macroscopic observation to mechanistic inquiry.

Around the year 2000, breakthroughs in molecular genetics and neuroimaging technologies catalyzed a profound methodological and conceptual transformation in PKU research. Using magnetic resonance spectroscopy, Sijens et al [70] provided the first direct evidence of metabolic alterations within cerebral white matter, challenging the long-standing assumption that neurological impairment was driven solely by circulating phenylalanine levels. Subsequent studies further delineated the association between cerebral metabolic patterns, executive function deficits, neurophysiological changes, and clinical severity, including analyses of large-scale brain network connectivity in adults with PKU. Currently, nutrition-focused research, exemplified by work from the European PKU Guideline Collaboration Group, integrates metabolomics and neuropsychological assessments to reveal how deficiencies in essential amino acids disrupt neurotransmitter synthesis and influence neurodevelopmental tendencies [71]. Together, these advances established “metabolic dysregulation affecting neurological function” as a core research paradigm and formed an interconnected knowledge framework that spans theoretical hypotheses, clinical observations, and mechanistic studies.

Findings from citation burst analysis indicate that the field is now undergoing a second paradigm shift, moving from mechanistic explanation toward clinical translation. Recent highly cited studies increasingly focus on the metabolic-brain axis, multi-omics integration, and precision nutrition strategies, reflecting the transformative impacts of high-throughput sequencing, metabolomics, and systems-level analytical platforms. With these technologies, researchers can characterize metabolic status in situ, map metabolic pathways, and model nutrient-metabolism-neural interactions in ways previously unattainable [62,71,72]. These advances are steering PKU research toward prevention-oriented metabolic management and precision medicine. Future priorities include the targeted restoration of key metabolic pathways to avert neurological injury, the integration of genetic and metabolic profiles for risk stratification, and the application of multi-omics approaches to identify therapeutic targets. Collectively, the progression from empirical observation to mechanistic understanding, and finally to translational innovation, reflects a maturing research paradigm. It suggests that metabolism-guided strategies will be at the forefront of future PKU management. In addition to dietary management, pharmacological treatment, and enzyme substitution therapy, gene therapy has emerged as a promising frontier in PKU research. Recent advances in adeno-associated virus–mediated gene delivery, genome editing technologies, and liver-directed therapeutic strategies have demonstrated encouraging preclinical and early clinical results. Although challenges related to long-term safety, durability of therapeutic effects, and large-scale clinical implementation remain, gene therapy has the potential to provide sustained metabolic correction and may fundamentally transform the treatment paradigm for PKU in the future [9,10].

### Limitations

This study has several limitations that should be acknowledged. First, the analysis was based exclusively on publications indexed in the WoSCC. Although WoSCC is widely recognized as a reliable source for bibliometric research, relevant studies indexed exclusively in other databases, such as PubMed, Scopus, or Embase, may have been omitted. Second, only English-language publications were included, which may have introduced language bias and resulted in the underrepresentation of research from non–English-speaking regions. Third, although bibliometric indicators such as publication counts and citation frequencies are useful for evaluating research productivity and academic influence, they do not directly reflect methodological quality, clinical significance, or scientific rigor. In addition, citation-based metrics may be influenced by factors such as self-citation and differences in citation practices across disciplines. Fourth, despite efforts to standardize author names, institutional affiliations, and country information, residual inconsistencies in database records may have affected certain collaboration and productivity analyses. Finally, because the literature search was conducted on September 13, 2025, the publication data for 2025 represent only a partial year and should therefore be interpreted with caution. Nevertheless, by integrating VOSviewer and CiteSpace and examining 25 years of scholarly output, this study provides a comprehensive overview of the evolution, collaboration patterns, and emerging research directions in PKU treatment research.

### Conclusions

This bibliometric analysis systematically delineates the development of PKU research over the past 25 years. The field has advanced from an initial stage grounded in metabolic control and dietary intervention toward a mechanistic era characterized by elucidation of the metabolic-neural axis, and is now transitioning into a precision-medicine paradigm that integrates tailored nutrition, enzyme replacement therapies, and targeted metabolic regulation. Global research output continues to be dominated by the United States and several European countries, with China emerging as an increasingly influential contributor. Co-authorship networks and institutional collaboration patterns reveal a cohesive core of long-standing international collaborations that have consistently propelled high-impact progress. Keyword evolution and reference burst analyses jointly highlight individualized metabolic management through integrated multi-omics as the current frontier of PKU research.

## Abbreviations

IF: impact factor
PAH: phenylalanine hydroxylase
PKU: phenylketonuria
WoSCC: Web of Science Core Collection
